# A Study on Determinants of COVID-19 Preventive Health Behaviors of Mothers with Young Children in South Korea

**DOI:** 10.3390/healthcare10102111

**Published:** 2022-10-21

**Authors:** Hye-Gyung An, Chae-Min Yoon

**Affiliations:** 1 Department of Nursing, Youngsan University, Yangsan-si 50510, Korea; 2 Department of Nursing, Changshin University, Changwon-si 51352, Korea

**Keywords:** COVID-19, mother, preventive health behavior, Korea, young children

## Abstract

This study is a descriptive research study conducted to identify factors that affect children of Korean mothers the coronavirus disease 2019 (COVID-19) preventive health behavior. It was confirmed that knowledge of COVID-19, maternal confidence, and risk perception of COVID-19 infection were related to the Preventive health behaviors of COVID-19 of Korean mothers with children. The subjects of this study were 191 mothers residing in Korea and raising children under the age of 5, and data were collected through an online questionnaire. We used the Google platform to fill out a questionnaire and collect data using a network sampling method from mothers who voluntarily participated in a survey at an online community meeting of mothers with young children. The collected data were analyzed using descriptive statistics, factor analysis, t-test, Pearson’s correlation analysis, and multiple regression analysis using IBM SPSS statistics 21.0 program. Preventive health behaviors of COVID-19 factor 1 are positively correlated with: mothers’ COVID-19 knowledge (r = 0.192, *p* < 0.01), confidence in infant care knowledge(r = 0.179, *p* < 0.05), and satisfaction with the role of mother(r = 0.351, *p* < 0.001). Negatively correlated with: unacceptable risk perception(r = −0.222, *p*< 0.01). Preventive health behaviors of COVID-19 factor 2 are positively correlated with: mothers’ COVID-19 knowledge (r = 0.166, *p* < 0.05), confidence in infant care knowledge(r = 0.179, *p* < 0.05), and satisfaction with the role of mother(r = 0.338, *p* < 0.001). Negatively correlated with: unacceptable risk perception(r = −0.205, *p* < 0.01). To strengthen COVID-19 preventive health behavior of Korean mothers with young children, it is suggested that education programs should be developed to provide accurate knowledge, increase maternal confidence, and improve the risk perception of COVID-19 infection.

## 1. Introduction

The coronavirus disease (COVID-19), that broke out in a Chinese city in December 2019, hit the world very fast. In March 2020, World Health Organization (WHO) declared COVID-19 as a pandemic. From July 2022 to present, the cumulative total of confirmed cases was about 526,000,000, and as many as 6,310,000 died in the world (as of 1 July 2022 at 00). In Korea, the cumulative total of confirmed cases was about 366,386, and the number of deaths reached about 2858. 79.6% of confirmed cases are concentrated in the metropolitan area, and the weekly average daily number of confirmed cases in Gyeongnam is 113.4, the number of infected children under the age of 5 has not been confirmed (as of 1 November 2021 at 00) [1]. To prevent the spread of COVID-19, countries in the world strive to develop vaccines and treatment drugs. In spite of that, the emergence of coronavirus mutation and the rise in the number of confirmed cases still people make feel anxious and fearful. Although quarantine control is made in the national dimension, COVID-19 spreads fast and mutated viruses appear continuously. As a result, it is difficult to return to a mundane life that people had prior to COVID-19.

The public’s fear of COVID-19 infection made mandate mask-wearing settled fast in their activities of daily living. Nevertheless, not all age groups can wear masks well. Under 48 months of age may experience breathing difficulties when wearing a mask, so the mask duty has been lifted [1]. With the development of COVID-19 vaccines, adults have been inoculated. Under age of 12 Korean, however, are free of mandatory vaccination, since safety of vaccination is not secured.

In Korea, the government and Korean Society of Pediatric Infectious Diseases recommend that unnecessary going-out and contact with others be minimized and that young children stay at home in order to prevent their COVID-19 infection [2]. Compared to adults, children in early young children have mild levels of COVID-19 infection frequency and severity, and their immunity system and function and their defense system for infection are not developed enough. In particular, they are vulnerable to respiratory infection [3]. Therefore, it is necessary to come up with separate infection prevention measures for these of young children. 

Since the outbreak of COVID-19, the government has limited contact activities and movement. During the pandemic, Korea raised its infectious disease crisis alert level from the alert level to the highest serious level, and the public care system was paralyzed due to the closure of public childcare system and educational institutions [4]. At that time, 73.3% of young children families who used daycare did not use public daycare system to protect their young children. In addition, 53.1% of families with young children had no experience of using the emergency daycare service, and the reason they did not use it was because of concerns about infection [5]. In the situation where COVID-19 directly and indirectly threatens human health, mothers, who are mainly in charge of raising young children, more frequently came to determine health behaviors for preventing the COVID-19 infection of their of young children. Mothers not only play a role in creating a hygienic health environment that can have a significant impact on the lifelong health of their children and other family members, but also play a major role in lifestyle decisions such as exercise and nutrition [6]. The health of infants can be directly affected by what mothers do to manage their children’s health [7]. Therefore, it is necessary to develop an effective maternal COVID-19 infection control education program for young children.

The health belief model (HBM) is a representative social cognitive theory that has the explanatory power to determine or predict people’s behavior for health, and is evaluated as a theory that can be widely applied in predicting people’s health preventive behavior [8]. HBM components include modification factors (e.g., knowledge, etc.), perceived threat and perceived expectation, which are individual beliefs, and cue to action. HBM is assumed that an individual’s behavior is determined by modifying factors, personal beliefs, and behavioral cues. This study based on the health belief model theoretically, using infection prevention knowledge as a modifying factor, perceived threat among personal beliefs as risk perception, and perceived expectation variable as maternal confidence, the purpose of this study is to identify the determinants of mother’s COVID-19 preventive health behavior.

### 1.1. Infection Prevention Knowledge and Maternal Confidence

The infection prevention knowledge that mothers care for young children directly influences their children’s infection preventive behaviors [9], and is a preceding factor maternal self-confidence [10]. Maternal confidence is the perception of the ability of mothers who care for theirs of young children, cognizes and responds to signals of young children, and are satisfied with their maternal roles [11]. According to the research by Russell [12] maternal confidence is categorized into confidence in the knowledge about child rearing, confidence in child rearing performance (tasks), and satisfaction with maternal roles (feelings). The more the mothers have maternal confidence, the more they have positive child-rearing behaviors and health promotion behaviors for children [13]. Therefore, this study tries to find whether the COVID-19 knowledge and maternal confidence of mothers with young children influence preventive health behaviors of COVID-19.

### 1.2. Risk Perception of COVID-19 Infection

Risk perception means one’s subjective thinking generated by the judgment of subjective value or feeling that one has in the situation before any specific damage arises as a harmful result [14]. HBM shows that risk perception influences health behaviors [15]. In the previous studies on COVID-19 risk perception and preventive behaviors of nurses, the more nurses had risk perception and COVID-19 knowledge, they more they had preventive behaviors [16,17]. As such, risk perception depends on the perception of pandemic situations like COVID-19. Such perception can become a critical variable of the preventive behavior intention of infectious diseases. But, there are no studies confirming that mothers with young children are aware of the Risk perception of COVID-19 infection on their young children’s infection prevention behavior. Therefore, it is necessary to investigate the mother’s perception of the risk of infection with COVID-19.

### 1.3. COVID-19 Infection Preventive Behaviors

The previous studies for the improvement in the performance of COVID-19 infection preventive behaviors reported that knowledge and attitudes significantly affected the performance of infection preventive behaviors [18,19]. The related studies in foreign countries also revealed that COVID-19 knowledge, attitudes, and perception had significant relations with infection preventive behaviors [20,21,22,23]. As such, there were many studies of the influential factors on adults’ COVID-19 infection preventive behaviors. However, it was hard to find any research on the determinants of the Preventive health behaviors of COVID-19 that mothers with young children had.

Therefore, this study tries to find the influential factors of the determinants of the preventive health behaviors of COVID-19 that mothers with young children. And to provide a fundamental material for developing the COVID-19 infection prevention education program to prevent COVID-19 infection of young children and improve their health. 

## 2. Materials and Methods

The purposes of this study are to analyze regressions between COVID-19 knowledge of mothers with young children, their maternal confidence, their risk perception, and their preventive health behaviors of COVID-19; to find the general characteristics of these study participants and their children, and their differences in COVID-19 knowledge, maternal confidence, perception of risk, Preventive health behaviors of COVID-19; to define the influential factors on preventive health behaviors of COVID-19; and thereby to provide a fundamental material for the education program of COVID-19 infection prevention for young children.

### 2.1. Participants and Data Collection

This study used a descriptive correlational design to explore the COVID-19 knowledge, maternal confidence, risk perception, and preventive health behaviors of COVID-19 that mothers with young children have, and to define regressions between variables.

The subjects of this study are mothers raising young children under 5 years of age, who understood the purposes of this study and voluntarily participated in research. Network sampling, which selects easily accessible cases as research subjects, was selected for the convenience of the researcher. The researcher used an online community of mothers created to share parenting information in Gyeongsangnam-do. The criteria to select the study participants were as follows: (1) Those who understand contents of the Korean questionnaire (2) Those who live in Gyeongsangnam-do province. At the time of data collection, the current status of COVID-19 infection in Korea was limited to the Gyeongsangnam-do region with a similar rate of spread because there was a big difference in the degree of regional infection. The questionnaire was completed online using the Google platform, and subjects were recruited through social media community advertisements. Instructions for participating in the survey were posted online (e.g., you can withdraw your participation at any time, benefits from participating in the survey, the Declaration of Helsinki has been implemented, etc.) and your consent to participate has been confirmed. The data collection period was 2 weeks from 15 October 2021 to 30 October 2021. With respect to the number of research participants, G * power 3.1.9 version program, the criteria for multiple linear regression analysis-effect size (f) 0.15, significance level (α) 0.05, power (1-β) 0.95, and the number of predictive variables 13-were applied. As a result, 189 participants were required. In consideration of the dropout rate 20%, a total of 227 respondents were obtained. Among them, 36 who had young children whose age fails to meet the criterion, and who gave inconsistent answers were excluded. Finally, 191 copies were used for analysis. To check the distribution of the collected data, skewness and kurtosis values were checked. Since it was a value between −2 and +2, the normal distribution was confirmed.

### 2.2. Statistical Analysis

The data collected in this study were analyzed using IBM SPSS statistics 21.0 program, and the statistical significance level was tested at 0.05. The general characteristics of the subjects were analyzed by frequency and percentage. The *t*-test, ANOVA, and Scheffe test were conducted for the difference in knowledge of COVID-19, maternal confidence, risk perception of COVID-19 infection, and preventive health behaviors of COVID-19 according to general characteristics.

Prior to the main analysis, exploratory factor analysis (EFA) was performed to test the construct validity of some of the variables used in this study, which were partially modified by the researcher to suit this study with the approval of the tool developer. Factor analysis is to identify factors that are not directly observed based on a set of observed variables, and the purpose is to simplify the contents by grouping numerous variables into a small number of factors [22]. By grouping each item into a few factors, it was possible to determine whether each item measures the same concept, that is, whether it is valid.

In this study, principal components analysis was performed as a factor extraction method, and only factors with values greater than or equal to the specified eigenvalue were extracted. For factor rotation, the orthogonal factor rotation method, verimax rotation, was performed. The results were derived by selecting a factor of 0.40 or higher, which is a normal acceptance criterion for factor loading, which indicates the degree of correlation between factors of each variable. In order to verify the reliability of the items measured by the item scale as composed of homogeneous items, the Cronbach’s α coefficient, which looks at the degree of inter-item consistency, was calculated.

Next, Pearson’s correlation analysis and multiple regression analysis were performed to identify the factors affecting the preventive behaviors of COVID-19, focusing on the classified factors.

### 2.3. Instruments

#### 2.3.1. Demographic Variables of Mother and Infant

In this study, the general characteristics of mothers, the subjects of this study, were six items including age, corona vaccination, underlying disease, occupation, and current health status. As a general characteristic of a child, it consists of three items: the child’s age, gender, and current health status.

#### 2.3.2. Knowledge of COVID-19

The knowledge of COVID-19 measurement tool developed by Kim and Cheon [24] was used. The researcher was authorized to use the tool. A total of 20 questions are treated as 1 point if they are correct and 0 points if they are wrong or do not know, ranging from 0 to 20 points. Content validity (CVI) was tested by one doctor, one professional nurse, and two nursing professors. The CVI at the time of tool development was 0.98, and the CVI in this study was 0.92.

#### 2.3.3. Maternal Confidence

The maternal confidence tool is a tool created by Chae [25] by translating the Maternal Confidence Questionnaire of Parker and Zahr [26]. This tool consists of a total of 14 items consisting of 12 positive items and 2 negative items (Nos. 10 and 12), and the range of possible scores on the Likert 5-point scale ranges from 14 to 70 points. Higher scores indicate higher maternal confidence. The tool was divided into sub-areas at the time of development by Parker and Zahr [26]. Since the tool modified by Chae [25] to apply to parents of premature infants was measured without subdomains, in this study, exploratory factor analysis was performed and sub-factors were reconfirmed. And the researcher gave it a name. As a result of the final analysis, the overall variance explanatory power of maternal confidence was 67.736%. As a result of the exploratory factor analysis based on previous studies, the sub-factors of confidence were named maternal confidence factor 1 (confidence in infant care knowledge), maternal confidence factor 2 (confidence in performing infant care), and maternal confidence factor 3 (satisfaction with the role of mother). When Parker and Zahr [26] developed the tool, Cronbach’s α = 0.89. In Chae [25]’s study, Cronbach’s α = 0.78, in this study the overall Cronbach’s α = 0.86, Factor 1 Cronbach’s α = 0.931, Factor 2 Cronbach’s α = 0.728, Factor 3 Cronbach’s α = 0.628. The reliability of factor 3 was less than the threshold of 0.70, but the overall tool reliability was above the threshold and included in the analysis because it is a factor that significantly influences the study results. These analyzes suggest that there may be limitations in generalizing the findings of the study.

#### 2.3.4. Risk Perception of COVID-19 Infection

In general, risk perception refers to people’s subjective perception of risk situations, and refers to attitudes or judgments about risk [27]. In this study, it refers to people’s subjective attitudes or judgments about the risk perception of COVID-19 infection. And the value measured using the tool developed for COVID-19 infection risk recognition by Han and Lee [28]. This tool is a 14-item Likert 5-point scale, with each question ranging from 1 point for ‘not at all’ to 5 points for ‘strongly agree’, with higher scores indicating higher risk perception of COVID-19 infection. Sung [29]’s tool referenced by Han and Lee [28] is a tool created by referring to the risk perception and risk characteristics of Marris, Langford, O’Riordan [30] and Slovic [27]. The risk perception tools of Marris, Langford and O’Riordan [30] and Slovic [27] were tools with subdomains. Therefore After obtaining permission from Han and Lee [28], this researcher analyzed and named sub-factors through exploratory factor analysis. As Questions 9 and 14 were removed from the COVID-19 risk perception tool through the scale purification process. The removed variable values had no effect on the statistics. As a result of the final analysis, the overall variance explanatory power of maternal confidence was 67.403%. As a result of the exploratory factor analysis based on previous studies, sub-factors of recognition were named risk perception of COVID-19 infection factor 1(General risk perception), risk perception of COVID-19 infection factor 2 (dread risk perception), and risk perception of COVID-19 infection factor 3(unacceptable risk perception). In the study of Sung [29], Cronbach’s α = 0.82, Han and Lee [28], Cronbach’s α = 0.85, and in this study, overall Cronbach’s α = 0.87, Factor 1 Cronbach’s α = 0.893, Factor 2 Cronbach’s α = 0.749, Factor 3 Cronbach’s α = 0.784.

#### 2.3.5. Preventive Health Behaviors of COVID-19

The mother’s Preventive health behaviors of COVID-19 was measured using the tool developed by Han and Lee [28], which modified and supplemented the respiratory infection prevention health behavior tool developed by Lee [31]. It is a 20-item Likert 5-point scale, ranging from 0 for ‘not at all’ to 4 for ‘strongly agree’, and the range of possible score ranges from 0 to 80 points. Higher scores indicate higher Preventive health behaviors of COVID-19. The tools of Han and Lee [28] were based on the tools of the respiratory infection prevention health behavior tool developed with subdomains by Lee [31]. So after asking the Han and Lee [28]’s permission, in this study, sub-factors were renamed and used through exploratory factor analysis. As a result of the exploratory factor analysis based on previous studies, sub-factors of recognition were named Preventive health behaviors of COVID-19 factor 1(Individual preventive health Behaviors), COVID-19 preventive health behaviors factor 2 (Collective preventive health behaviors). As a result of the final analysis, the overall variance explanatory power of Preventive health behaviors of COVID-19 was 72.184%. In the study of Han and Lee [28], Cronbach’s α = 0.86, and in this study, overall Cronbach’s α = 0.95, Factor 1 Cronbach’s α = 0.966, Factor 2 Cronbach’s α = 0.940.

## 3. Results

### 3.1. Demographic Variables of Mother and Infant

Table 1 shows the participants’ general characteristics. The average age of mothers was 37.58 years, with 83.8% of those vaccinated against COVID-19. Those who were not vaccinated were 16%. The current state of health perceived by the mother was poor 13.1%, average 79.6%, Good 7.3%. The average age of the children was 2.13 years, and the gender was 42.4% male and 57.6% female. Current health status is poor 16.8%, average 59.7%, Good 23.6%. There were significant differences in knowledge of COVID-19 and Maternal confidence according to the mother’s perceived current health status. In Scheffe’ post-mortem analysis, knowledge of COVID-19 was significantly higher in good and moderate than in poor (F = 3.09, *p* < 0.001). There was a significant difference in maternal confidence according to the current health status of the young children perceived by the mother (F = 8.88, *p* < 0.001). In Scheffe’ post-mortem analysis, maternal self-confidence was significantly higher in average than in poor and good than moderate (Table 1).

### 3.2. Correlation between Mother’s and Young Children’s Current Health Status, Knowledge, Maternal Confidence, Risk Perception, Preventive Health Behaviors of COVID-19

Pearson correlation analysis was performed to examine the relationship between knowledge, maternal confidence, risk perception, preventive health behaviors of COVID-19, mother’s and young children’s current health status (Table 2).

First, the mother’s current health status is the child’s current health status (r = 0.430, *p* < 0.01), knowledge of COVID-19 (r = 0.238, *p* < 0.01), maternal confidence factor 2, a sub-factor of maternal confidence (r = 0.294, *p* < 0.01), positive correlation existed. The mother’s current health status was negatively correlated with unacceptable risk perception a sub-factor of risk perception (r = −0.143, *p* < 0.05). Second, the children’s current health status is maternal confidence factor 1 (r = 0.236, *p* < 0.01), factor 2 (r = 0.210, *p* < 0.01), factor 3 (r = 0.430, *p* < 0.05) and there was a positive correlation. Third, preventive health behaviors of COVID-19 factor 1, a sub-factor of behavior, had a positive correlation with knowledge (r = 0.192, *p* < 0.01), maternal confidence factor 1 (r = 0.179, *p* < 0.05), and maternal confidence factor 3 (r = 0.351, *p* < 0.001), a sub-factor of maternal confidence. And there was a negative correlation with risk perception factor 3 (r = −0.222, *p* < 0.01), which is a sub-factor of risk perception of COVID-19. In addition preventive health behaviors of COVID-19 factor 2, a sub-factor of preventive health behaviors of COVID-19, had a positive correlation with knowledge of COVID-19 (r = 0.167, *p* < 0.05), maternal confidence factor 1 (r = 0.166, *p* < 0.05), and maternal confidence factor 3 (r = 0.338, *p* < 0.001), there was a positive correlation. And there was a negative correlation with risk perception factor 3 (r = −0.205, *p* < 0.01), which is a sub-factor of risk perception of COVID-19.

### 3.3. Effect of Mother’s and Young Children’s Current Health Status, COVID-19 Knowledge, Maternal Confidence, and Risk Perception on Preventive Health Behaviors of COVID-19

Multiple regression analysis was performed to examine the effects of COVID-19 knowledge, maternal confidence, and risk perception on Preventive health behaviors of COVID-19. First, in order to check the multi-collinearity of the explanatory variables knowledge, confidence, and perception, the tolerance and variance inflation factor (VIF) were examined. As a result, the tolerance was 0.664~0.841 and the variance expansion factor was 1.301~1.506. It was found that collinearity does not exist.

#### 3.3.1. Effect of Mother’s and Young Children’s Current Health Status, COVID-19 Knowledge, Maternal Confidence, and Risk Perception on Preventive Health Behaviors of COVID-19 Factor 1

Multiple regression analysis was performed to examine the effects of mother’s and young children’s current health status, COVID-19 knowledge, maternal confidence, and risk perception on Preventive health behaviors of COVID-19 factor 1 (Table 3). As a result, the explanatory power (R2) of the multiple regression model was 0.164, which could explain 16.4% of the behavior factor 1 using COVID-19 knowledge, maternal confidence, and risk perception. It was confirmed that this multiple regression model was statistically significant (F = 5.153, *p* < 0.001).

Also, looking at the effects of COVID-19 knowledge, maternal confidence, and risk perception on behavior factor 1, it can be seen that confidence factor 3 (β = 0.280, *p* < 0.001) affects Preventive health behaviors of COVID-19 factor 1. In other words, it can be seen that as the degree of maternal confidence factor 3 increases, Preventive health behaviors of COVID-19 factor 1 also increases.

#### 3.3.2. Effect of Mother’s and Young Children’s Current Health Status, COVID-19 Knowledge, Maternal Confidence, and Risk Perception on Preventive Health Behaviors of COVID-19 Factor 2

Multiple regression analysis was performed to examine the effects of mother’s and young children’s current health status, COVID-19 knowledge, maternal confidence, and risk perception on Preventive health behaviors of COVID-19 factor 2 (Table 4). As a result, the explanatory power (R2) of the multiple regression model was 0.125, which could explain 12.5% of the behavior factor 2 using COVID-19 knowledge, maternal confidence, and risk perception. It was confirmed that this multiple regression model was statistically significant (F = 4.022, *p* < 0.001).

Also, looking at the effects of COVID-19 knowledge, maternal confidence, and risk perception on behavior factor 2, it can be seen that confidence factor 3 (β = 0.288, *p* < 0.001) affects Preventive health behaviors of COVID-19 factor 2. That is, it can be seen that as the degree of maternal confidence factor 3 increases, Preventive health behaviors of COVID-19 factor also increases, and as the degree of risk perception factor 3 decreases, Preventive health behaviors of COVID-19 factor increases. Also, through the standardization coefficient, it can be seen that the maternal confidence factor 3 (β = 0.288) has more influence on the Preventive health behaviors of COVID-19 factor than the risk perception factor 3 (β = −0.169).

## 4. Discussion

In order to investigate the factors affecting preventive health behaviors of COVID-19, it was divided into Individual preventive health behaviors (factor 1) and Collective preventive health behaviors (factor 2). This is because, unlike factor 1, factor 2 was judged to be difficult to control with only the individual will to act.

First, as a result of analyzing the correlation with major variables according to general characteristics, it was found that the better the mother’s current health status, the higher the knowledge of COVID-19, maternal performance confidence (maternal confidence sub-factor 2), and the young children’s current health status. Also, the lower the mother’s unacceptable risk perception (risk perception sub-factor 3), the better the mother’s current health status. These results are similar to Yi, Jung and Han [32] that showed that the higher the positive attitude, the higher the implementation of infection prevention behavior.

Second, it was found that the better the young children’s current health status, the higher the maternal confidence. Knowledge of COVID-19 and risk perception of COVID-19 were not significantly related to the young children’s current health status. Based on these results, the researcher intends to interpret that in order to improve the health of the young children, it is necessary to increase maternal confidence above all else. Therefore, in order to strengthen the preventive health Behavior of COVID-19 for mothers with young children against infectious diseases, education content that can improve maternal confidence should be included.

Third, as a result of multiple regression analysis to investigate the effect on Individual preventive health Behavior of COVID-19, the explanatory power of mother’s and young children’s current health status, knowledge of COVID-19, maternal confidence, and risk perception of COVID-19 was 16.4%. As for the effect on collective prevention health behavior of COVID-19, mother’s and young children’s current health status, knowledge of COVID-19, maternal confidence, and risk perception of COVID-19 were explanatory 12.5%. The reason why the individual preventive health behavior of COVID-19 was greater is interpreted because collective prevention health behavior cannot be improved by individual beliefs or health status alone. Contrary to the study Yi, Jung and Han [32] that said that the level of knowledge was not related to the performance of infection prevention behavior, this study was a factor influencing the preventive health behavior of COVID-19. These results are similar to those of Choi [33] and Abdel [34].

According to the health belief model, an individual’s beliefs are affected by modifying variables such as current health status and knowledge. Knowledge of new infectious diseases such as COVID-19 is also an important modifying variable criterion for personal beliefs that perceive the risk of infectious diseases as a perceived threat [35]. Therefore, in order to strengthen preventive health behaviors of COVID-19, it can lead to changes in risk perception of COVID-19 and maternal confidence, which are personal beliefs, by providing knowledge education that includes up-to-date and accurate disease information. However, in this study, mothers’ educational needs were not investigated. In a future study, it is suggested to assess the mother’s educational needs.

Fourth, it was confirmed that the maternal confidence sub-factor 3 had a greater effect on the individual preventive health Behavior of COVID-19 than the risk perception sub-factor 3 through the standardization coefficient. According to Lee et al. [36], it was found that mother’s mental health had more influence on young children’s health prevention behavior than other groups. During the coronavirus pandemic, there have been many studies examining negative mental health factors such as anxiety [37], psychological instability [38], harsh parenting [39], and lack of emotional care [40] among mothers with young children during the COVID-19 pandemic. Maternal confidence is an important factor that positively influences preventive health behaviors. Therefore, this study is significant in confirming that positive factors are factors influencing preventive behavior.

From these results, the researcher proposes the development of an effective educational program to improve the health preventive behavior of mothers with young children and to deliver the latest knowledge and improve maternal confidence.

## 5. Conclusions

This study investigated general characteristics, knowledge of COVID-19, maternal confidence, risk perception of COVID-19 infection, and Preventive health behaviors of COVID-19 in order to identify factors influencing the preventive behaviors against COVID-19 targeting mothers with young children. The results of this study are meaningful in that they provide basic data for the development of health care education programs for mothers of infants and toddlers in the event of a future outbreak of a pandemic infection.

According a result, as the unacceptable risk perception (risk perception of COVID-19 infection factor 3) was lower, the collective COVID-19 prevention behavior increased. Also, it was found that there was a significant correlation Preventive health behaviors of COVID-19 between mothers’ knowledge of COVID-19, maternal confidence, and risk perception of COVID-19. In particular, the higher the satisfaction with the role of mother (maternal confidence factor 3), the more Individual preventive health Behaviors of COVID-19 for their children increased. However, in factor analysis, the maternal confidence factor 3 is less than the absolute confidence value of 0.70, so there is a big limitation in generalizing the results. Nevertheless, the researchers argue that education to boost maternal confidence is necessary to get mothers to do many COVID-19 preventive actions for their children. Since maternal confidence and risk perception are, after all, areas that correspond to individual beliefs, education is necessary to correctly form personal beliefs. 

In addition, if the mother’s knowledge level is checked and information is provided to prevent the rapidly spreading respiratory infection, it will be possible to promote the health of the children as well as the health of the family. For follow-up studies, research to apply and evaluate various interventions that can increase knowledge, confidence, and risk awareness is suggested.

## Figures and Tables

**Table 1:** General characteristics and research variables of participants (*N* = 191).

Characteristics	Categories	n (%) or M ± SD	Knowledge		Maternal Confidence		Risk Perception of COVID-19 Infection		COVID-19 Preventive Health Behaviors	
Mother			M ± SD	t or F (*p*)	M ± SD	t or F (*p*)	M ± SD	t or F (*p*)	M ± SD	t or F (*p*)
				Scheffe		Scheffe		Scheffe		Scheffe
Age (year)		37.58 ± 3.91								
COVID-19 vaccination	Yes	160 (83.8)	0.62 ± 0.11	0.05 (0.825)	3.93 ± 0.54	0.01 (0.944)	3.77 ± 0.53	0.08 (0.773)	4.11 ± 0.82	0.05 (0.831)
	No	31 (16.0)	0.63 ± 0.10		3.94 ± 0.51		3.74 ± 0.43		4.14 ± 0.85	
Presence of underlying diseases	Yes	18 (9.4)	0.61 ± 0.09	0.28 (0.598)	4.05 ± 0.36	1.12 (0.290)	3.88 ± 0.55	1.03 (0.311)	4.03 ± 1.00	0.22 (0.639)
	No	173 (90.6)	0.63 ± 0.11		3.92 ± 0.55		3.75 ± 0.51		4.12 ± 0.80	
Occupation	Unemployed	88 (46.1)	0.63 ± 0.10	0.07 (0.788)	3.94 ± 0.51	0.11 (0.740)	3.70 ± 0.46	2.39 (0.124)	4.08 ± 0.87	0.34 (0.562)
	Employed	103 (53.9)	0.62 ± 0.11		3.92 ± 0.56		3.82 ± 0.55		4.15 ± 0.78	
Current health status	Good ^a^	14 (7.3)	0.63 ± 0.07	9.71 (<0.001)a,b > c	3.61 ± 0.37	3.10 (0.047)	3.72 ± 0.48	0.36 (0.699)	4.10 ± 0.83	1.19 (0.307)
	Moderate ^b^	152 (79.6)	0.64 ± 0.09		3.94 ± 0.50		3.78 ± 0.50		4.15 ± 0.78	
	Poor ^c^	25 (13.1)	0.54 ± 0.17		4.04 ± 0.75		3.70 ± 0.59		3.88 ± 1.05	
**Young children**										
Age (year)		2.13 ± 2.27								
Gender	Male	81 (42.4)	0.61 ± 0.11	3.21 (0.075)	3.90 ± 0.56	0.35 (0.556)	3.75 ± 0.50	0.11 (0.739)	4.17 ± 0.80	0.66 (0.418)
	Female	110 (57.6)	0.64 ± 0.10		3.95 ± 0.52		3.77 ± 0.52		4.07 ± 0.83	
Current health status	Good ^a^	45 (23.6)	0.62 ± 0.15	1.91 (0.151)	4.14 ± 0.60	8.88 (<0.001) a > b > c	3.77 ± 0.56	0.41 (0.664)	4.09 ± 1.04	0.28 (0.759)
	Moderate ^b^	114 (59.7)	0.64 ± 0.10		3.92 ± 0.49		3.74 ± 0.51		4.15 ± 0.76	
	Poor ^c^	32 (16.8)	0.60 ± 0.08		3.64 ± 0.50		3.83 ± 0.44		4.03 ± 0.70	

a, b, c are described in current health status.

**Table 2:** Correlation between mother’s and young children’s current health status, knowledge, maternal confidence, risk perception, preventive health behaviors of COVID-19.

	(1) Mother’s Current Health Status	(2) Young Children’s Current Health Status	(3) Knowledge	(4) Maternal Confidence			(5) Risk Perception			(6) Preventive Health Behaviors	
	1	2	3	4-1	4-2	4-3	5-1	5-2	5-3	6-1	6-2
1. (1)	1										
2. (2)	0.430 **	1									
3. (3)	0.238 **	0.039	1								
2. (4) factor 1	0.075	0.236 **	0.360 ***	1							
3. (4) factor 2	0.294 **	0.210 **	0.074	0.387 ***	1						
4. (4) factor 3	0.049	0.179 *	0.168 *	0.182 *	−0.079	1					
5. (5) factor 1	0.054	0.035	0.141	0.186 **	0.187 **	−0.110	1				
6. (5) factor 2	0.006	−0.059	0.180 *	0.260 ***	0.255 ***	−0.129	0.507 ***	1			
7. (5) factor 3	−0.143 *	−0.007	0.009	−0.011	0.298 ***	−0.253 ***	0.313 ***	0.257 ***	1		
8. (6) factor 1	0.128	0.009	0.192 **	0.179 *	−0.141	0.351 ***	0.032	−0.064	−0.222 **	1	
9. (6) factor 2	0.015	−0.039	0.167 *	0.166 *	−0.047	0.338 ***	0.049	0.058	−0.205 **	0.819 ***	1
M ^1^	1.94	1.93	12.53	4.26	3.30	3.41	4.03	4.04	3.36	4.19	4.04
S.D ^2^	0.449	0.632	2.159	0.653	0.743	0.960	0.706	0.727	0.982	0.920	0.802

^1^ M: Mean, ^2^ SD: Standard Deviation, *: *p* < 0.05, **: *p* < 0.01, ***: *p* < 0.001.

**Table 3:** Effect of knowledge, maternal confidence, and risk perception on Preventive health behaviors of COVID-19 factor 1.

	B	S.E	*β*	*t*	*p*	Tolerance	VIF
(constant)	2.228	0.709	-	3.143	0.002	-	-
Mather’s current health status	0.115	0.164	0.056	0.701	0.484	0.683	1.464
Young children’s current health status	0.056	0.111	0.038	0.502	0.616	0.749	1.335
knowledge	0.036	0.032	0.085	1.119	0.265	0.768	1.301
Maternal confidence factor 1	0.221	0.115	0.157	1.931	0.055	0.664	1.506
Maternal confidence factor 2	−0.158	0.099	−0.127	−1.596	0.112	0.691	1.447
Maternal confidence factor 3	0.269	0.069	0.280	3.873 ***	0.000	0.841	1.189
Risk perception factor 1	0.155	0.104	0.119	1.493	0.137	0.691	1.446
Risk perception factor 2	−0.101	0.102	−0.080	−0.990	0.323	0.681	1.469
Risk perception factor 3	−0.113	0.071	−0.121	−1.593	0.113	0.765	1.307
R^2^	adj. R^2^			*F*		*p*	
0.204	0.164			5.153 ***		0.000	

*** *p* < 0.001.

**Table 4:** Effect of COVID-19 knowledge, maternal confidence, and risk perception on Preventive health behaviors of COVID-19 factor 2.

	B	S.E	*β*	*t*	*p*	Tolerance	VIF
(constant)	2.426	0.632	-	3.839 ***	0.000	-	-
Mather’s current health status	−0.085	0.147	−0.048	−0.583	0.561	0.683	1.464
Young children’s current health status	0.056	0.099	0.044	0.562	0.575	0.749	1.335
knowledge	0.032	0.029	0.086	1.110	0.268	0.768	1.301
Maternal confidence factor 1	0.087	0.102	0.071	0.854	0.394	0.664	1.506
Maternal confidence factor 2	−0.051	0.088	−0.048	−0.584	0.560	0.691	1.447
Maternal confidence factor 3	0.241	0.062	0.288	3.899 ***	0.000	0.841	1.189
Risk perception factor 1	0.088	0.093	0.077	0.946	0.345	0.691	1.446
Risk perception factor 2	0.089	0.091	0.080	0.979	0.329	0.681	1.469
Risk perception factor 3	−0.138	0.063	−0.169	−2.181 *	0.030	0.765	1.307
R^2^	adj. R^2^			*F*		*p*	
0.167	0.125			4.022 ***		0.000	

* *p* < 0.05, *** *p* < 0.001.

## Data Availability

Not applicable.
